# Tumor Accumulation and Off-Target Biodistribution of an Indocyanine-Green Fluorescent Nanotracer: An Ex Vivo Study on an Orthotopic Murine Model of Breast Cancer

**DOI:** 10.3390/ijms22041601

**Published:** 2021-02-05

**Authors:** Marta Sevieri, Leopoldo Sitia, Arianna Bonizzi, Marta Truffi, Serena Mazzucchelli, Fabio Corsi

**Affiliations:** 1 Dipartimento di Scienze Biomediche e cliniche “L. Sacco”, Università di Milano, 20157 Milan, Italy; marta.sevieri@unimi.it (M.S.); leopoldo.sitia@unimi.it (L.S.); arianna.bonizzi@unimi.it (A.B.); 2 Istituti Clinici Scientifici Maugeri IRCCS, 27100 Pavia, Italy; marta.truffi@icsmaugeri.it

**Keywords:** ferritin nanoparticles, ICG, tumor targeting, nanomedicine, ex vivo imaging, fluorescence-guided surgery, fluorescence

## Abstract

Indocyanine green (ICG) is a near infrared fluorescent tracer used in image-guided surgery to assist surgeons during resection. Despite appearing as a very promising tool for surgical oncology, its employment in this area is limited to lymph node mapping or to laparoscopic surgery, as it lacks tumor targeting specificity. Recently, a nanoformulation of this dye has been proposed with the aim toward tumor targeting specificity in order to expand its employment in surgical oncology. This nanosystem is constituted by 24 monomers of H-Ferritin (HFn), which self-assemble into a spherical cage structure enclosing the indocyanine green fluorescent tracer. These HFn nanocages were demonstrated to display tumor homing due to the specific interaction between the HFn nanocage and transferrin receptor 1, which is overexpressed in most tumor tissues. Here, we provide an ex vivo detailed comparison between the biodistribution of this nanotracer and free ICG, combining the results obtained with the Karl Storz endoscope that is currently used in clinical practice and the quantification of the ICG signal derived from the fluorescence imaging system IVIS Lumina II. These insights demonstrate the suitability of this novel HFn-based nanosystem in fluorescence-guided oncological surgery.

## 1. Introduction

Fluorescence-guided surgery (FGS) is an intraoperative medical procedure that provides real-time fluorescence images of the operating field [1]. It is gaining interest in surgical oncology due to its potential to improve tumor margin visualization and the identification of tumor deposits. Indeed, a more precise anatomic localization of cancer tissues during resection would be crucial for the success of any oncological surgery and decisive in maximizing the benefits for patients [2,3,4].

The implementation of FGS requires the development of more accurate and sensitive imaging devices and of an effective fluorescence contrast agent [5,6]. Among different probes that may assist this technique, indocyanine green (ICG) is one of the most used and well known [1,7,8,9], despite other near-infrared (NIR) fluorophores being approved by the Food and Drug Administration (FDA), including Methylene Blue, 5-Aminolevulinic acid (5-ALA), and Fluorescein, which are also suitable for FGS applications but are much less frequently employed [10,11].

ICG is a tricarbocyanine, water-soluble fluorescent dye with substantial fluorescence emission in the near-infrared (NIR) wavelength region (700–900 nm) [8,12,13,14]. Due to its fluorescent characteristics and safety, ICG is currently used for several cancer-related surgical applications, including sentinel lymph node (SLN) mapping, the identification of solid tumors, lymphography, angiography, and anatomical imaging during surgery [14,15,16,17,18]. However, since it lacks specific tumor targeting and suffers from rapid degradation and blood-stream elimination [19,20], the potential for its use in oncological FGS still has certain limitations [3,21,22].

ICG is a passive tumor-targeted probe and its performance in ensuring the unambiguous identification of cancer tissue is still modest and insufficient in providing a trustworthy exploitation of this technique [1]. Therefore, most research focuses on designing nanocarriers as delivery systems for ICG with the aim of tackling some of its current issues and to expand its possible applications in cancer diagnosis and treatment [22,23]. Hence, the overall goal would be to develop systems with high specificity for tumors able to provide enhanced contrast between cancer tissues or affected lymph nodes and healthy tissue, in order to tailor specific surgeries [22,24,25].

In the last 15 years, several studies have been carried out using ferritin bio-nanoparticles [26]. These nanoparticles, thanks to their protein nature, show an excellent biocompatibility profile, great solubility in biological fluids and good stability at high temperatures and in the presence of denaturing agents [27,28]. H-Ferritin (HFn) nanoparticles appear as cave spheres consisting of 24 monomers of human ferritin H chains with an external diameter of 12 nm.

They demonstrate a natural homing toward cancer cells due to the specific recognition of the transferrin receptor-1 (TfR1), which is overexpressed in all tumor subtypes and represents a universal molecule for tumor targeting as its expression in cancer is higher than that seen in other healthy cells [29,30,31]. Their physiological features and their capacity to encapsulate drugs or fluorescent probes, makes ferritin nanocages ideal platforms for oncological applications, such as drug delivery and diagnostics [32].

Several HFn-based nanodrugs have been proposed for drug delivery with excellent results in terms of specific tumor recognition and increased activity with lower side effects [33,34,35]; however, ferritin nanoparticles suggested for diagnostic purposes are restricted to magnetic resonance imaging [27,32,36] and there are only a few examples of optical imaging applications [37] that do not include FGS or ICG exploitation. In light of this, we have proposed ICG loaded HFn nanocages as an in vivo system for FGS that will allow the surgeon to perform a more accurate surgical resection of the tumor with the ultimate goal of improving surgical outcomes [38].

Preliminary studies have demonstrated in vitro the tumor-targeted recognition of ICG upon nanoformulation in HFn nanocages. These studies have demonstrated that nanoformulation also affects the fluorescence stability, improving it, and resulting in better fluorescence signal in tumors [38]. Here, we performed an ex vivo analysis of HFn-ICG tumor accumulation and biodistribution to better elucidate the differences in ICG behavior in vivo upon nanoformulation.

## 2. Results and Discussion

### 2.1. HFn-ICG Displayed a Higher Intratumor Accumulation Compared to Free ICG

To further study the suitability of HFn-ICG as tumor-targeted nanotracer for in vivo image-guided surgery [38], we decided to perform an ex vivo study on a syngeneic orthotopic murine model of breast cancer. Starting from the in vivo pilot experiment that provided evidence regarding the potential of HFn−ICG to target the tumor mass [38], in this study, we evaluated the tumor accumulation and the biodistribution of both free and nano-formulated ICG ex vivo with the aim to demonstrate the improved performances and the potential of HFn-ICG as a nano-tracer for fluorescence tumor detection.

HFn displayed natural tumor homing due to its capability to specifically bind the transferrin 1 receptor (TfR1) and be internalized through receptor-mediated endocytosis. These features have been fully explored in tumor-targeted drug delivery, using HFn to treat different kinds of cancers. Many drugs have been encapsulated and tested, including Olaparib, Everolimus, Cis-Platinum, Curcumin, and Mitoxantrone [26,39,40,41,42,43], and the most interesting results were obtained with doxorubicin [33,34,35,44,45,46].

Despite the HFn-based nanosystems being applied to cancer detection were well studied with positron emission tomography application, magnetic resonance, and multimodal imaging, their application in fluorescence image-guided surgery is almost unexplored [38]. We used a model of murine breast cancer, obtained by the injection of 4T1 cells into the mammary fat pad of *Balb/C* female mice. Tumor-bearing mice were divided into two experimental groups and injected with 3.8 mg/kg of free ICG or nano-formulated ICG. After 6 and 24 h mice were sacrificed, the tumors were collected and imaged by the KARL STORZ NIR/ICG endoscopic system, as reported in Video S1–S5.

**Figure 1:**
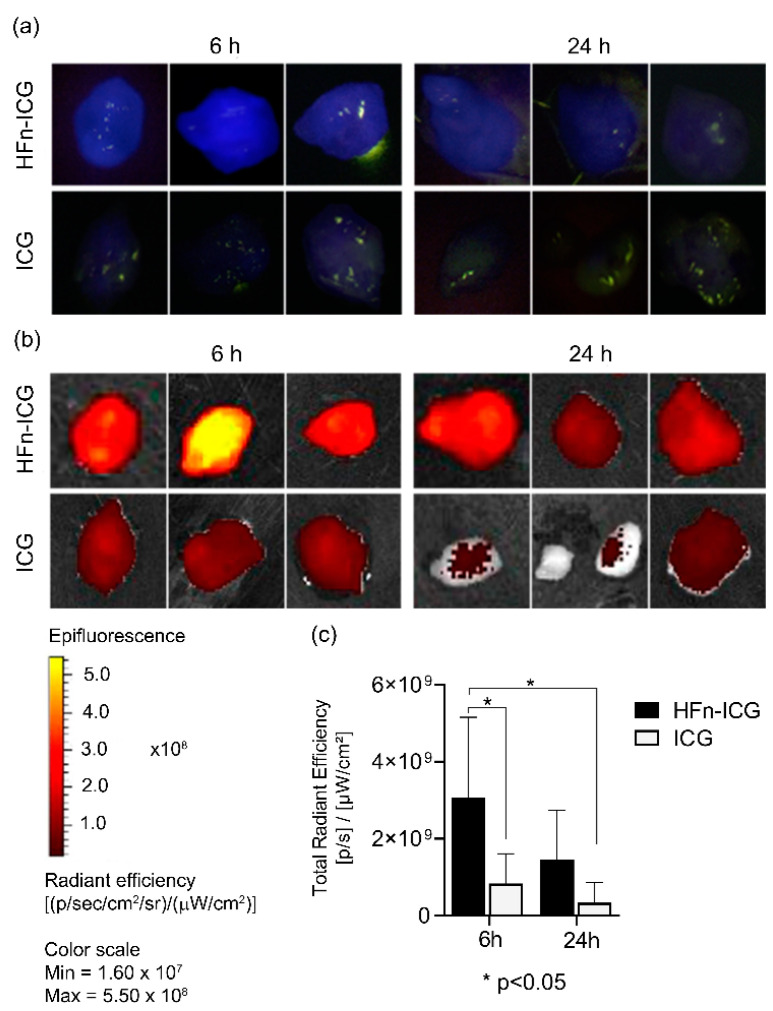
The tumor targeting of H-Ferritin (HFn)−indocyanine green (ICG) and free ICG was evaluated in 4T1 tumor-bearing mice, 6 and 24 h after intravenous administration with a KARL STORZ near-infrared (NIR)/ICG endoscopic system ((**a**), blue signal) and a IVIS Lumina II system (**b**). In (**a**,**b**) images of three representative tumors of each group are shown. Imaging analysis of data obtained by IVIS Lumina II allowed us to quantify the dye in the tumor (**c**). The fluorescence was higher in HFn−ICG-treated mice than in free ICG-treated mice at both 6 and 24 h. There is a statistical significance between HFn-ICG and free ICG at 6 h (*p*-value = 0.0345) and between HFn-ICG at 6 h and free ICG detected at 24 h (*p*-value = 0.0112). Color scale expressed as the total radiant efficiency (×10^8^), *n* = 6. * *p* < 0.05.

This represents one of the main systems used in in vivo surgery and allowed us to really test the suitability of our nanoconstruct. As shown in Figure 1a, an intense blue fluorescent signal was localized in the tumor mass of mice treated with HFn-ICG at 6 h, while mice injected with ICG displayed a barely noticeable signal, more similar to a dark blue shade. At 24 h after injection, the tumors harvested from mice treated with HFn-ICG showed a fluorescent signal lower than the one detected at 6 h but still evident, while, in the group injected with free ICG, the fluorescence signal was hardly visible (Supplementary Material, video S2–S4).

To confirm and obtain a quantitative analysis of these observations, we coupled the imaging from the KARL STORZ NIR/ICG endoscopic system to the imaging performed by the IVIS Lumina II system, which allowed us also to quantify the fluorescence due to the software for analysis. The IVIS lumina II acquisitions corroborated that the HFn-ICG tumor accumulation at 6 h was still visible at 24 h, while the free ICG did not accumulate and was rapidly cleared (Figure 1b,c). Indeed, there was a significant difference in the levels of fluorescence between HFn-ICG and free ICG at 6 h as well as with ICG at 24 h. Therefore, in mice injected with HFn-ICG it was possible to detect a higher fluorescence signal compared to the signal visible at 6 h for free ICG.

### 2.2. HFn Encapsulation Improves Tumor Uptake of ICG

**Figure 2:**
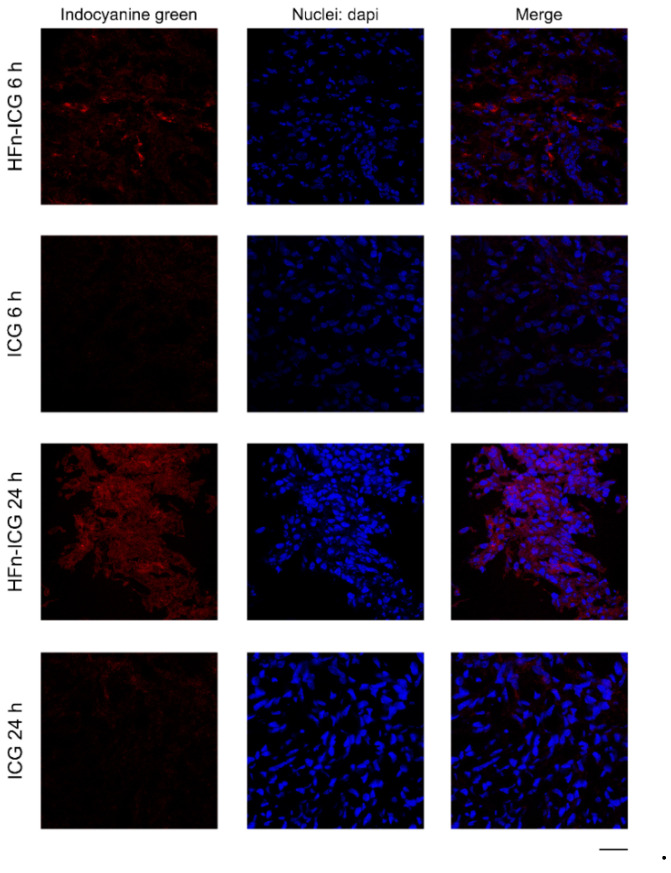
Confocal images of tumor cryosections collected from mice injected with HFn−ICG or free ICG (red) and sacrificed at 6 and 24 h. Nuclei were stained with 4’,6-Diamidino-2-phenylindole dihydrochloride (dapi; blue). Scale bar = 50 μm.

To assess if the higher accumulation in cancer was really due to improved HFn-ICG performances related to a better uptake of the ICG, we performed a histological evaluation of tumors collected at 6 and 24 h from both groups to localize the signal. We obtained confocal microscopy images of the tumor (Figure 2), where the red signal associated with ICG was higher in samples treated with the nanoformulation and showed a different distribution compared to that observed with free ICG both at 6 and at 24 h.

**Figure 3:**
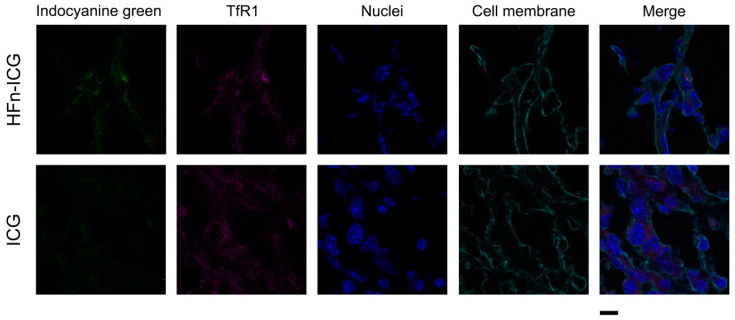
Colocalization between the transferrin receptor-1 (TfR1) receptor and ICG upon HFn-mediated internalization. Tumors collected from mice injected with HFn−ICG or free ICG (green) and sacrificed at 6 h, were cryosectioned and labelled to study the colocalization with TfR1 (purple). Nuclei and cell membranes were stained with dapi (blue) and Wheat Germ Agglutinin-Alexa Fluor 488 (cyan), respectively. Scale bar = 10 μm.

Different to the dotted distribution of intracellular HFn−ICG that is due to the vesicle-mediated uptake mechanism, as demonstrated by the colocalization between the ICG signal and TfR1 reported in Figure 3, the uptake pattern observed for free ICG was less intense and specific (Figure 2 and Figure 3). This might be due to the specific and low uptake of free ICG in cancer cells and to a fast degradation of the molecule that leads to a fluorescence loss. In light of this, HFn encapsulation could significantly improve the intracellular uptake of ICG and preserve its fluorescence, as already previously suggested [38], therefore ensuring a more precise identification of the tumor.

### 2.3. HFn Encapsulation Markedly Improved the ICG Kinetics of Biodistribution

**Figure 4:**
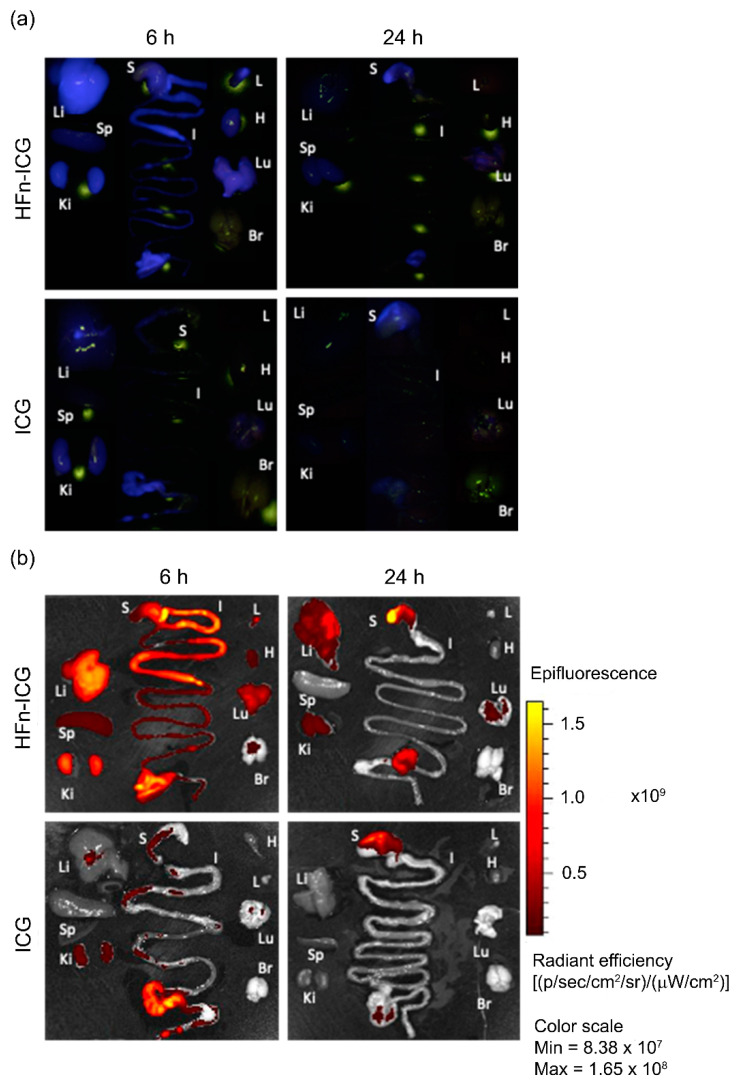
Representative images of organs collected from tumor-bearing mice, 6 and 24 h after I.V. administration of HFn-ICG and free ICG obtained with the KARL STORZ NIR/ICG endoscopic system (**a**, blue signal) and IVIS Lumina II system (**b**). In each panel, it is possible to observe the following organs: Liver (Li), Spleen (Sp), Kidneys (Ki), Stomach (S), Intestine (I), Lymph node (L), Heart (H), Lung (Lu), and Brain (Br)). Color scale expressed as the total radiant efficiency (×10^9^).

As previously described with the tumor analysis, the major organs were also examined by the KARL STORZ NIR/ICG endoscopic system to evaluate the off-target biodistribution of fluorescence in each district (Figure 4a). In this case, there was also a significant difference between the mice injected with HFn-ICG and free ICG. HFn-ICG was detectable at 6 h in the tumor (Figure 1a, blue signal) and also at the liver, kidneys, and at the gastrointestinal tract. Additionally, even with a lower intensity, it is observed at the axillary lymph nodes, heart, and lungs.

This biodistribution profile is consistent with the ICG metabolism. This occurs in the liver, where it is accumulated into bile salts and released in the intestines, allowing its excretion with feces. As expected, when ICG is administered as a free dye it is rapidly metabolized. A weak fluorescence signal was observed at 6 h in the liver and in the last part of the intestinal tract. At 24 h after injection, there was a noticeable decrease in the fluorescence levels compared to the 6 h time point. However, in the organs from mice injected with free ICG, there was an almost complete washout of the dye, while in those from mice injected with HFn-ICG, the fluorescence signal (blue) was still present in the liver, kidneys, stomach, and in the distal part of the gut, in addition to the already discussed accumulation at the tumor (Figure 1a).

While the detection of the ICG signal in the gut, liver, and kidneys was consistent with its metabolism, the signal in the stomach was surprising, as the HFn-ICG was administered by parenteral injection. However, the reason was easily attributable to the sphincter relaxation occurring upon sacrifice that allowed the diffusion of ICG-rich bile salts into the stomach. These results overall suggested the crucial role of the HFn nanocage in protecting ICG from rapid metabolism and degradation, which instead represents the destiny of the free dye.

**Figure 5:**
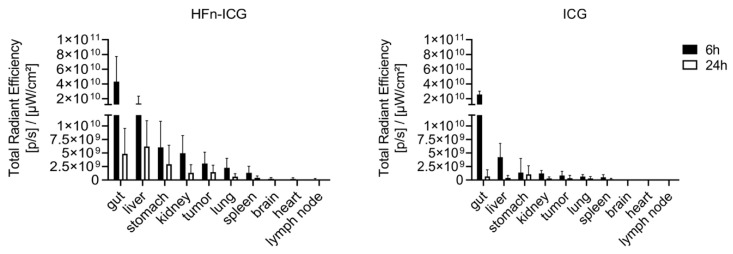
Imaging analysis of data obtained by IVIS Lumina II allowed us to quantify the signal associated with ICG by drawing regions of interest (ROIs) around each of the individual tissues. The histograms show the mean value measured in each organ of the mice treated with HFn-ICG (left panel) and with free ICG (right panel) and sacrificed 6 and 24 h after the injection. The bars are the mean value ± standard deviation (SD), *n* = 6.

Figure 4b shows an overview of the distribution of the signal associated with ICG in the same organs observed in Figure 4a. These representative imaging scans performed by the IVIS Lumina II reflect what is reported by Figure 4a and the analysis of the imaging scans described in Figure 5 reveal a striking difference between the two formulations in terms of the kinetics of biodistribution. ICG is rapidly metabolized and it was unable to accumulate specifically at any organ.

At 6 h, as previously mentioned, when administered as a free dye, ICG was found in the liver and the gut but was then promptly excreted as no fluorescent signal is visible at either location at 24 h. On the contrary, HFn-ICG allowed us to detect higher signals in all the organs and appeared to preserve the dye’s fluorescence up to 24 h in the gut, liver, stomach, and at the tumor, which is our focus.

**Figure 6:**
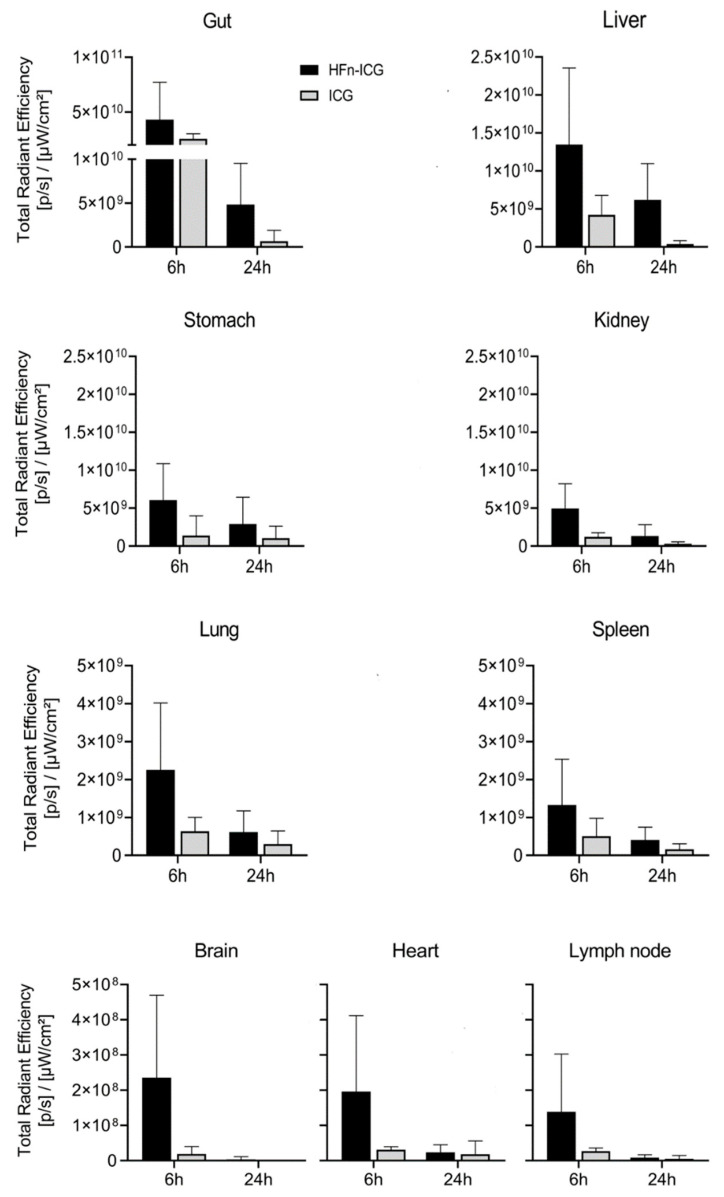
Imaging analysis of the ICG signal obtained by IVIS Lumina II and associated to different organs. Quantification of the signal associated with ICG was performed by drawing ROIs around each of the individual tissues. The histograms show the mean value measured in each organ of mice treated with HFn-ICG (**left** panel) and with free ICG (**right** panel) and sacrificed 6 and 24 h after the injection. The bars are the mean value ± SD, *n* = 6.

In Figure 6, we report the quantification of the signal for each individual organ. The gut, which exhibited the highest signal compared to the other organs, displayed, at 6 h, an accumulation of ICG comparable between the two formulations. Afterward, in both groups, there was a progressive decay of the signal. However, in mice treated with HFn-ICG, the presence of the dye was persistent at 24 h. The fluorescence signal in the gut was not located in the intestinal wall but was only restricted to the chyle, and then to the feces. In the liver, it was possible to see an accumulation of the signal in favor of HFn-ICG still visible at the last time point and more intense compared to the free ICG.

Presumably, ICG, which has a quicker clearance, accumulated in the liver at an earlier time and, at 6 h, was already completely excreted into the bile (Figure 6). The fluorescence intensity registered in the stomach had a similar trend to that in the kidney in both groups. The fluorescence signal appeared attenuated at 24 h for HFn-ICG, while it was visible at lower levels for ICG even at 6 h. With regard to the lungs, spleen, brain, heart, and axillary lymph nodes, the signal was far lower, with an order of magnitude of 10^8^–10^9^, compared to the fluorescence at the gut, liver, stomach, and kidney (10^10^–10^11^).

The fluorescence signal of HFn-ICG was higher compared to the free ICG at each time point in every organ of this second group. In the brain, heart, and lymph nodes, the fluorescence of ICG was hardly observable. Overall, the ability of targeting the tumor mass with improved fluorescence accumulation in tumor, as confirmed by this ex vivo study, makes HFn−ICG a powerful system for the delivery of ICG. Further studies with mass spectrometry should be performed to elucidate if the increased fluorescence signal observed in cancer tissue and also in off-target organs is due to a better profile of the biodistribution or to the increased stability of the fluorescence signal acquired upon nanoformulation.

## 3. Materials and Methods

### 3.1. Development of ICG-Loaded-HFn Nanoparticles

HFn was purchased from MoLiRom s.r.l. (Rome, Italy). The ICG was nano-formulated exploiting the ability of HFn to disassemble and reassemble its quaternary structure in response to changes in the pH, as previously reported [38].

### 3.2. Animals

The animals were managed according to procedures approved by the Italian Ministry of Health (Protocol Number 611/2019-PR, 6 August 2019). All procedures involving animals and their health were conducted in accordance with the 3R principles to minimize the number of mice used and their collateral suffering. The animals were housed in specific pathogen-free conditions and were kept in cages with free access to water and food.

### 3.3. Tumor Targeting and Biodistribution

For the biodistribution studies, we recruited six animals for each experimental time point. Seven-week-old female BALB/c mice were injected into the mammary fat pad with 100,000 4T1-Luc cells (Bioware Ultra, PerkinElmer, Waltham, MA, USA). After 21 days, the mice were intravenously injected in the tail vein with ICG or HFn−ICG at a concentration of 3.8 mg/Kg. Subsequently, the mice were sacrificed by cervical dislocation at 6 or 24 h to follow the biodistribution of the two administered formulations. Immediately after the sacrifice, we performed an accurate autopsy by means of the KARL STORZ NIR/ICG endoscopic system (OPAL1 Technology, equipped with a high-end full HD camera IMAGE 1 SPIES and a xenon light source D-light P SCB; KARL STORZ GmbH & Co. KG, Tuttlingen, Germany).

This allowed us to detect the fluorescent signal observable in blue at the tumor site and in terms of the organ distribution. The tumor and the major organs, i.e., the liver, stomach, gut, kidneys, spleen, hearth, lung, brain, and lymph nodes were collected and imaged with an IVIS Lumina II imaging system (PerkinElmer, Waltham, MA, USA). Ex vivo scans of organs were performed with the following acquisition parameters: Excitation filter: 745 nm, emission filter: ICG, exposure time: 2 s, binning factor: Medium, f/Stop: 2, Field of View: D.

The Living Image Software 4.3.1 (Perkin Elmer, Waltham, MA, USA) conjugated with the Image Math tool was used to separate the ICG signal from the tissue autofluorescence, image processing, and fluorescence signal quantification analysis. In detail, the Image Math tool offered together with Living Image^®^ 4.3.1 software allowed the subtraction of the tissue autofluorescence background from the signal. Due to the acquisition of an imaging scan with a background filter (i.e., green fluorescent protein filter), it was possible to separate the ICG signal from the tissue autofluorescence and perform the correct image processing and quantification. Tables reporting Signal to noise (SNR) and Signal to Background (SBR) ratios have been provided as Supplementary Material (Tables S1 and S2, respectively). Finally, all the tumors were frozen at −80 °C for cryosectioning and histological analysis.

### 3.4. Confocal Laser Scanning Microscopy

Cryosections of 9 μm were obtained from each 4T1 tumor and, after adhesion in glass slides, were counterstained with 0.2 μg/mL dapi (4′,6-diamino-2-phenylindole; Thermo Fisher Scientific Inc., Waltham, MA, USA) for 10 min at room temperature, washed thrice, and mounted with Prolong Gold (Life technology; #P10144, Thermo Fisher Scientific Inc., Waltham, MA, USA). Microscopy analyses of cryosections were performed with a Leica SP8 microscope confocal system equipped with lasers at 405, 488, 513, and 633 nm (Leica, Wetzlar, Germany). Tumor images were acquired at a 512 × 512 pixel resolution using a 63× immersion oil lens.

To assess the colocalization between nanoformulated ICG and TfR1, we labelled the tumor cryosection as follows. The cryosections were air dried at room temperature (RT) for 15 min, rinsed with phosphate saline buffer (PBS), and fixed for 5 min with 2% paraformaldehyde (Sigma-Aldrich, Merck Life Science, Milano, Italy). They were then permeabilized for 10 min at RT with 0.1% Triton X-100 (Sigma-Aldrich, Merck Life Science, Saint Louis, MO, USA) in PBS. Afterward, the samples were incubated for 1 h at RT with a solution containing 2% Bovine Serum Albumin (BSA; Sigma-Aldrich, Merck Life Science, Saint Louis, MO, USA)) and 2% goat serum (Euroclone, Pero, Italy) in PBS. TfR1 labelling was performed with the anti-TfR1 antibody (1:200; ab84036; Abcam, Cambridge, UK) by overnight incubation at 4 °C.

After three washes in PBS, the anti-TfR1 antibody was recognized by Alexa Fluor 488-conjugated antibody against rabbit Immunoglobulins G (IgGs; Thermo Fisher Scientific Inc., Waltham, MA, USA) at a 1:300 dilution by incubating for 2 h at RT in PBS, 2% BSA, 2% goat serum, and 0.2 μg/mL dapi (4′,6-diamino-2-phenylindole; Thermo Fisher Scientific Inc., Waltham, MA, USA). Finally, the samples were counterstained with wheat germ agglutinin and Alexa Fluor™ 488 Conjugate incubating for 1 h at RT in PBS (1:200; W11261; Thermo Fisher Scientific Inc., Waltham, MA, USA) for visualization of the cell membranes. A single plane image of the tumor section was acquired at a 512 × 512 pixel resolution using a 63× immersion oil lens and applying a digital zoom of three times.

### 3.5. Statistical Analysis

All data were expressed as the mean ± SD. Student’s *t*-test and the *p* values were evaluated using the GraphPad Prism version 6.00 for Windows (Graph-Pad Software, San Diego, CA, USA). The sample size was calculated referring to [47], using the Power and Sample Size Calculator program with a statistical power of 80% and an alpha error of 5% calculated.

## 4. Conclusions

Intraoperative visualization of tumors by means of fluorescence-guided surgery (FGS) may not only allow more accurate tumor resections but also improve safety by reducing unnecessary damage to normal tissues with benefits for both the surgeons and cancer patients. Therefore, practical methods for improving the surgeon’s ability to resect tumors are needed. Here, we provided a strict comparison in terms of the tumor accumulation, off-target biodistribution and kinetics of clearance prodromic to in vivo assess tracking capability of HFn-ICG. These results support the suitability of HFn-ICG for the FGS application; however, our future endeavors will be focused on the improvement of the HFn circulation time in order to maximize its capability to localize at a tumor for longer to thereby allow its application in other kinds of tumors.

## Data Availability

Data available in a publicly accessible repository https://doi.org/10.13130/RD_UNIMI/ZKBQFS, after publication.

## Supplementary Materials

Supplementary materials can be found at https://www.mdpi.com/1422-0067/22/4/1601/s1. Video S1. Non-treated mouse imaged by the KARL STORZ NIR/ICG endoscopic system. The ICG signal is absent, and the tissue autofluorescence is evidenced by green light. Video S2. Mouse injected with HFn-ICG and imaged after 24 h by the KARL STORZ NIR/ICG endoscopic system in vivo. This video displays how the fluorescent signal of ICG, visible in blue, allowed us to easily detect the tumor mass and was clearly distinguishable from the background, which is visualized in green. Video S3. Mouse injected with HFn-ICG and imaged after 24 h by the KARL STORZ NIR/ICG endoscopic system during the surgical intervention. Here, it is possible to observe the labelled tumor mass during the surgical intervention. The tumor mass is clearly distinguishable from the surrounding tissue. Video S4. Mouse injected with free ICG and imaged after 24 h by the KARL STORZ NIR/ICG endoscopic system during the surgical intervention. Here, it is possible to observe the labelled tumor mass during the surgery. The tumor mass of the mouse injected with free ICG does not appear as fluorescent. Video S5. Mouse injected with HFn-ICG and imaged after 2 h by the KARL STORZ NIR/ICG endoscopic system. Here, it is possible to see the ICG signal before the achievement of complete organ distribution. Indeed, until 2 h after injection, the ICG, free or nanoformulated, is mainly present in the bloodstream, resulting in a blurred visualization of the anatomical compartments. Table S1. SNR obtained from the ratio between the mean total radiant efficiency of signal acquired with ICG filter and the mean total radiant efficiency of signal acquired with GFP filter (*n* = 6). Table S2. SBR obtained from the ratio between the mean total radiant efficiency of signal acquired with ICG filter in a ROI drawn on target organ and the mean total radiant efficiency of signal acquired with ICG filter in a ROI drawn on background (*n* = 6).

## Abbreviations

ICG	Indocyanine Green
HFn	H-Ferritin
HFn−ICG	H-Ferritin loaded with ICG
FGS	Fluorescence-guided surgery
FDA	Food and Drug Administration
NIR	Near-infrared
SLN	Sentinel lymph node
TfR1	Transferrin receptor 1
DAPI	4’,6-Diamidino-2-phenylindole dihydrochloride
ROI	Region of interest
SD	Standard deviation
PBS	Phosphate saline buffer
BSA	Bovine serum albumin
IgG	Immunoglobulin G
SNR	Signal to noise ratio
SBR	Signal to background ratio
