# Supplementary material for: Tumor Accumulation and Off-Target Biodistribution of an Indocyanine-Green Fluorescent Nanotracer: An Ex Vivo Study on an Orthotopic Murine Model of Breast Cancer
Source: Int J Mol Sci. 2021 Feb 5;22(4):1601. doi: 10.3390/ijms22041601 (PMC7915532; doi:10.3390/ijms22041601)
Supplement: Supplementary file 1 [file ijms-22-01601-s001.zip › SI_Sevieri.docx]

*Article*

Tumor Accumulation and off-Target Biodistribution of an Indocyanine-Green Fluorescent Nanotracer: An Ex Vivo Study on an Orthotopic Murine Model of Breast Cancer

M. Sevieri ^1^, L. Sitia ^1^, A. Bonizzi ^1^, M. Truffi ^2^, S. Mazzucchelli ^1,^* and F. Corsi ^1,2,^*

^1^ Dipartimento di Scienze Biomediche e cliniche “L. Sacco”, Università di Milano, Milan, 20157, Italy;
marta.sevieri@unimi.it (M.S.); leopoldo.sitia@unimi.it (L.S.); arianna.bonizzi@unimi.it (A.B.)

^2^ Istituti Clinici Scientifici Maugeri IRCCS, Pavia 27100, Italy;; [marta.truffi@icsmaugeri.it](mailto:marta.truffi@icsmaugeri.it) (M.T.).

***** Correspondence: serena.mazzucchelli@unimi.it (S.M.); fabio.corsi@unimi.it (F.C.)

**Supporting Information**

**Table S1.** SNR obtained from the ratio between the mean total radiant efficiency of signal acquired with ICG filter and the mean total radiant efficiency of signal acquired with GFP filter (n=6).

**Table S2.** SBR obtained from the ratio between the mean total radiant efficiency of signal acquired with ICG filter in a ROI drawn on target organ and the mean total radiant efficiency of signal acquired with ICG filter in a ROI drawn on background (n=6).
