# Supplementary material for: Tumor Accumulation and Off-Target Biodistribution of an Indocyanine-Green Fluorescent Nanotracer: An Ex Vivo Study on an Orthotopic Murine Model of Breast Cancer
Source: Int J Mol Sci. 2021 Feb 5;22(4):1601. doi: 10.3390/ijms22041601 (PMC7915532; doi:10.3390/ijms22041601)
Supplement: Supplementary file 1 [file ijms-22-01601-s001.zip › SI_Sevieri.pdf]

Article

Tumor Accumulation and off-Target Biodistribution of an Indocyanine-Green Fluorescent Nanotracer: An Ex Vivo Study on an Orthotopic Murine Model of Breast Cancer

M. Sevieri [1], L. Sitia [1], A. Bonizzi [1], M. Truffi [2], S. Mazzucchelli [1,*] and F. Corsi [1,2,*]

1 Dipartimento di Scienze Biomediche e cliniche "L. Sacco", Università di Milano, Milan, 20157, Italy;
marta.sevieri@unimi.it (M.S.); leopoldo.sitia@unimi.it (L.S.); arianna.bonizzi@unimi.it (A.B.)
2 Istituti Clinici Scientifici Maugeri IRCCS, Pavia 27100, Italy;; marta.truffi@icsmaugeri.it (M.T.).
* Correspondence: serena.mazzucchelli@unimi.it (S.M.); fabio.corsi@unimi.it (F.C.)

Supporting Information

Table S1. SNR obtained from the ratio between the mean total radiant efficiency of signal acquired with ICG filter and the mean total radiant efficiency of signal acquired with GFP filter (n=6).

	HFn-ICG 6H	ICG 6H	HFn-ICG 24H	ICG 24H
TUMOR	2,29E+01	1,74E+00	1,03E+01	2,38E+00
LIVER	1,87E+01	7,66E+00	9,24E+00	6,95E-01
SPLEEN	2,21E+01	6,29E+00	3,73E+00	2,53E+00
KIDNEYS	3,49E+01	1,03E+01	9,78E+00	2,35E+00
STOMACH	5,67E+00	1,35E+00	2,96E+00	5,88E-01
GUT	8,19E+00	4,37E+00	1,01E+00	1,30E-01
LYMPH NODES	1,23E+01	2,45E+00	8,94E-01	2,98E-01
HEART	5,16E+00	9,06E-01	7,91E-01	4,84E-01
LUNG	2,21E+01	7,07E+00	3,48E+00	3,30E+00
BRAIN	1,29E+00	1,71E-01	1,96E-02	0,00E+00

Table S2. SBR obtained from the ratio between the mean total radiant efficiency of signal acquired with ICG filter in a ROI drawn on target organ and the mean total radiant efficiency of signal acquired with ICG filter in a ROI drawn on background (n=6).

	HFn-ICG 6H	ICG 6H	HFn-ICG 24H	ICG 24H
TUMOR	1,77E+01	4,84E+00	9,75E+00	1,96E+00
LIVER	7,77E+01	2,44E+01	3,58E+01	2,19E+00
SPLEEN	7,67E+00	2,93E+00	2,34E+00	9,23E-01
KIDNEYS	2,86E+01	7,04E+00	7,63E+00	1,74E+00
STOMACH	3,49E+01	8,16E+00	1,62E+01	6,02E+00
GUT	2,48E+02	1,48E+02	2,76E+01	3,79E+00
LYMPH NODES	1,13E+00	1,79E-01	1,37E-01	1,04E-01
HEART	1,30E+01	3,67E+00	3,54E+00	1,72E+00
LUNG	1,35E+00	1,64E-01	1,93E-02	0,00E+00
BRAIN	8,00E-01	1,55E-01	5,01E-02	3,11E-02
